# Retraction: Disulfiram chelated with copper inhibits the growth of gastric cancer cells by modulating stress response and Wnt/β-catenin signaling

**DOI:** 10.3389/fonc.2024.1475033

**Published:** 2024-08-27

**Authors:** Frontiers Editorial Office

The journal retracts the 21 December 2020 article cited above.

Following publication, concerns were raised regarding the validity of the data in the article. The authors failed to provide the raw data and a satisfactory explanation during the investigation, which was conducted in accordance with Frontiers’ policies. Given the concerns about the validity of the data, and the lack of raw data, the editors no longer have confidence in the findings presented in the article.

This retraction was approved by the Chief Executive Editor of Frontiers. The authors agreed with the retraction.

